# The Implementation of an mHealth Intervention (ReZone) for the Self-Management of Overwhelming Feelings Among Young People

**DOI:** 10.2196/11958

**Published:** 2019-05-02

**Authors:** Chloe Edridge, Jessica Deighton, Miranda Wolpert, Julian Edbrooke-Childs

**Affiliations:** 1 Anna Freud National Centre for Children and Families London United Kingdom; 2 Clinical, Educational and Health Psychology University College London London United Kingdom

**Keywords:** cluster trial, behavioural difficulties, schools, mHealth, digital, mental health, mobile phone

## Abstract

**Background:**

The association between mental health difficulties and academic attainment is well established. There is increasing research on mobile health (mHealth) interventions to provide support for the mental health and education of young people. However, nonadoption and inadequate implementation of mHealth interventions are prevalent barriers to such trials.

**Objective:**

The aim of this study was to bridge this gap and examine the implementation of an mHealth intervention, ReZone, for young people in schools.

**Methods:**

Preliminary data for 79 students collected as part of a larger trial were analyzed. We additionally conducted postimplementation consultations with teachers.

**Results:**

ReZone was used 1043 times by 36 students in the intervention arm during the study period. Postimplementation teacher consultations provided data on implementation strategies, barriers, and facilitators.

**Conclusions:**

Implementation strategies, barriers, and facilitators for digital interventions need to be considered to limit nonadoption and inadequate implementation in larger trials. Important considerations involve tailoring the characteristics of the intervention to the requirements of the intended user group, the technology itself, and the organization in which it is implemented.

**Trial Registration:**

International Standard Randomised Controlled Trial Number: 13425994; http://www.isrctn.com/ISRCTN13425994

## Introduction

Recent evidence suggests that the levels of mental health difficulties in young people are increasing; for example, 1 in 4 young women experience emotional problems, including anxiety and depression [1]. The previous protocol published [2] for this study highlighted the prevalence of behavioral problems in young people and its association with academic attainment and mental health difficulties [3-6].

Mobile health (mHealth) provides new opportunities to support young people’s mental health. About 95% of adolescents aged 13-17 years own or have access to a smartphone [7]. Moreover, 69% of schools in the United Kingdom are currently using tablet computers [8].

The authors have co-designed an mHealth intervention, ReZone, with young people, parents/carers, and teachers. As described in detail previously [2], ReZone is based on cognitive behavioral therapy, mindfulness, and attention bias–modification training. These methods have been shown to be effective in addressing thought processes [9,10], self-regulation of attention [11], behavior and emotions [12,13], and negative cognitive biases [14,15].

However, a limitation of the existing technology-enabled interventions for young people is that the content of many of these interventions is not grounded in psychological theory or evidence-based practice [16]. There is a need for evidence from rigorous trials regarding the effectiveness of digital interventions for mental health among young people [17] in addition to research investigating the ways to best integrate these interventions into support provision [18].

Implementation and sustained adoption of interventions within schools can be impacted by a range of contextual issues. The general organizational structure, level and quality of support available, leadership, and administrative resources are factors of successful implementation of such interventions [19,20]. A wider system view must be adopted to consider the whole school in terms of alignment of the intervention with the school’s philosophy, goals, and policies [19]. The role, views, knowledge, and skills of the staff member who is most often at the forefront of implementation within the school must also be considered [21]. Teachers play a crucial role in the successful implementation of digital technologies, and their beliefs regarding teaching practices impact the level of resistance or acceptability of new technologies within the school [22].

Challenges are further highlighted in a recent evidence framework proposed for theorizing and evaluating nonadoption, abandonment, scale-up, spread, and sustainability (NASSS) of health and care technologies [23]. The NASSS framework outlines seven areas included for prediction and evaluation of the success of technology-supported health or social care programs. Areas for consideration are the condition or illness, the technology, the value proposition (ie, benefits of the intervention), the adopter system (comprising professional staff, patient, and lay caregivers), the organization(s), the wider context (institutional and societal), and the interaction and mutual adaptation between all these domains over time. Within these domains, there are potential challenges to consider, each classified as simple (few straightforward, predictable components), complicated (multiple interacting components or issues), or complex (dynamic, unpredictable, not easily disaggregated into constituent components).

The aim of this research was to bridge the gap in evidence of the implementation of mHealth interventions and examine the implementation of one such intervention, ReZone, for young people in schools. We report preliminary data from a cluster randomized trial of ReZone within schools. We additionally conducted postimplementation consultations with teachers to examine their views on and experiences of the facilitators and barriers to implementation.

## Methods

Further details on the methodology of the full randomized controlled trial (RCT) can be found in the published protocol [2]; this trial was pre-registered in a clinical trial registry (ISRCTN 13425994).

### Recruitment and Setting

Two types of schools were involved in the study: alternative provision primary/secondary schools and mainstream primary schools. Alternative provision schools are settings that provide education for young people who are unable to engage in mainstream education due to emotional or behavioral issues. All students within the participating classes were invited by their school to take part in this study. The preliminary data used in this paper relate to 10 clusters (classes), across six rural and urban schools (two primary and four alternative provision) within the United Kingdom.

### Participants and Procedure

At the time of manuscript preparation, 10 classes were recruited (four mainstream and six alternate provision), resulting in a sample of 79 students with a mean (SD) age of 11.14 (1.31) years (58% male). We lost 20 students to follow-up, yielding an attrition rate of 25%. All students (aged 10-15 years) in the schools participating in the project were eligible to participate in the study. Participant demographics are shown in Table 1 for the RCT dataset discussed in this paper. Postimplementation consultations were carried out with eight teachers from participating schools.

**Table 1:** Demographic characteristics (N=79).

Characteristic		Statistic
Age (years), mean (SD)		11.14 (1.31)
Male, n (%)		46 (58)
Alternate provision schools, n (%)		33 (42)
English as first language, n (%)		68 (86)
**Ethnicity, n (%)**		
	White	20 (25)
	Mixed	14 (18)
	Asian	36 (46)
	Black	8 (10)
	Other	1 (1)

The University College London Research Ethics committee provided ethics approval (number: 7969/001), and the study adhered to the relevant ethical guidelines (eg, the British Psychology Society [24]). This research is reported in line with Consolidated Standards of Reporting Trials (CONSORT) guidelines [25].

### Intervention

**Figure 1:**
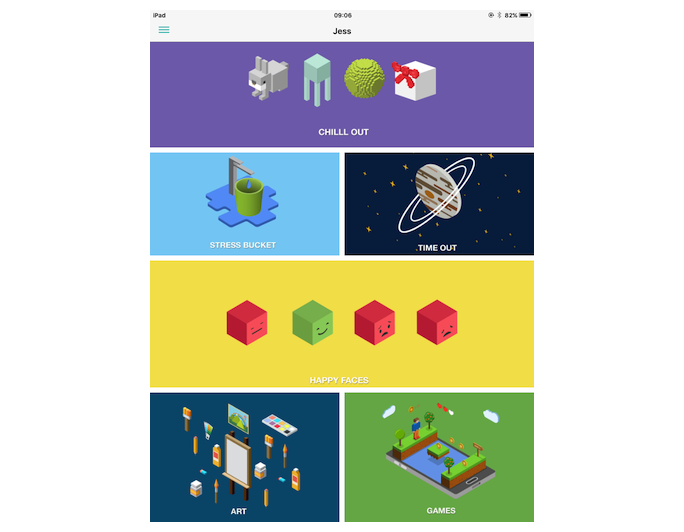
ReZone home screen.

Figure 1 presents the ReZone home screen. Textbox 1 presents the features of ReZone according to the guidelines for reporting mHealth interventions [26].

**Main features of ReZone.**

**Figure 2:**
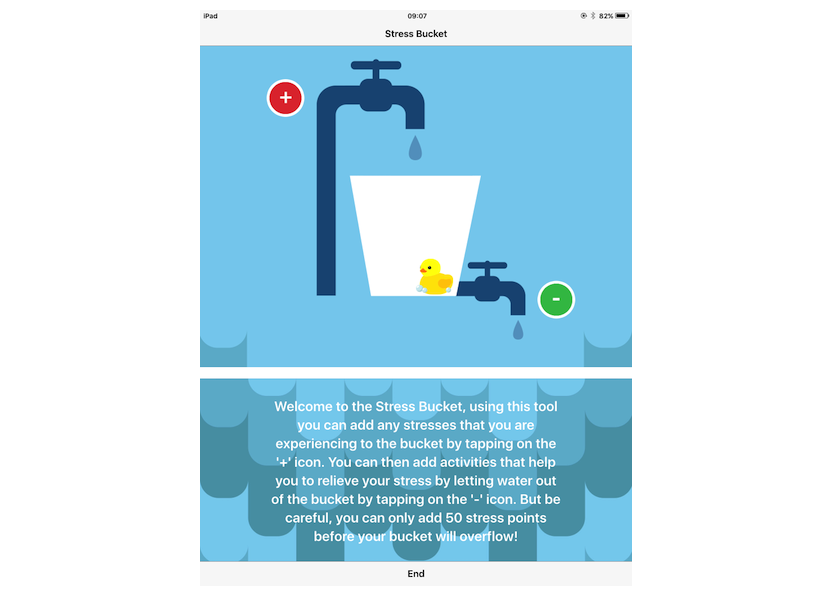
The stress bucket feature.

**Stress bucket:** The stress bucket lets the user add any stressors that he/she is experiencing to a bucket. The user is then able to introduce activities that help them cope with each stressor. The user can see the water in the bucket rise and fall as he/she adds and relieves stressors, respectively. If the bucket reaches 50 stress points, it will overflow (Figure 2).

**Timeout:** Timeout asks the user to think through a time when he/she felt stressed, angry, or upset. On the app, the user then works through all the events that led up to feeling this way and the following events that occurred. The user can also think through what he/she could have done differently to help the situation as a behavioral plan. The visualization is a rocket, and each thought process creates a cloud.

**Figure 3:**
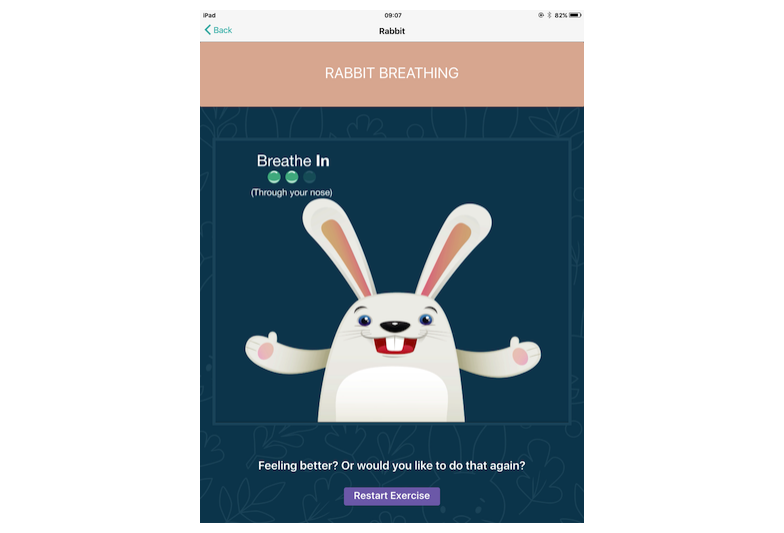
The breathing exercise.

**Chill out:** Chill out uses breathing to help the user calm down and relax. Each chill out activity is based around an object or animal (ie, rabbit, jellyfish, ball, or square) and uses breathing in different ways (Figure 3).

**Art therapy:** The user can choose between a castle, dinosaur, fish, goat, heart, helicopter, unicorn, rocket, footballer, sea, or turtle to color in. There is a range of colors and utensils to complete the drawing.

**Happy faces:** The user is given 30 seconds to find as many happy faces as he/she can amongst other faces depicting negative emotions.

**Game:** There is a game of “balloon blast” on the app. The user taps the screen to move the balloon up, trying to avoid all the obstacles, as hitting one will end the game. This game was created to provide a break or reward for the user in between the other features.

### Analytic Strategy

The usage data for this sample were collected and aggregated across all users, reporting the number of times each element of the tool was used. The postimplementation consultations were analyzed using framework analysis with determined topics and are discussed in line with the NASSS framework.

## Results

### Usage Statistics

Over the period of data collection, 1082 usage sessions were conducted for the 36 students in the intervention arm. In terms of usage statistics, the game (n=308), breathing exercises (n=276), and happy faces (n=241) were used the most; stress bucket (n=117), art (n=81), and time out (n=20) were used the least.

### Postimplementation Consultations

Consultations with teachers identified implementation strategies and barriers and facilitators as listed below.

#### Implementation Strategies

Teachers described two main implementation strategies for ReZone. Responsive use was the most common strategy, where teachers embedded ReZone into current tools to be used when students are feeling overwhelmed or a specific incident has occurred (eg, exams). The second strategy was preventative use where ReZone was used at set time points during the day (eg, start of lessons, tutor session, and break times) or in the morning to address any issues from the previous night and to inoculate against stressors that may arise during the day. Some teachers in mainstream schools reported that ReZone was available to students at all times on request, as part of a specified rota or other mental health support services offered (such as one-to-one support). Individual use of ReZone was preferred over whole-class use by some teachers in order to better tailor its use to individual needs and avoid influence or distraction of peers.

Following these overarching strategies, teachers described a combination of teacher-directed and student-led strategies that were often used. If use was completely led by students without any direction from the teacher, ReZone was not likely to be used. Nevertheless, some teachers extensively directed use at the beginning, but over time, transitioned control to students who asked to use ReZone.

#### Barriers and Facilitators to Implementation

Teachers described the pivotal role of school culture on the implementation of ReZone. In particular, teachers felt that incorporating the app into daily routines was more challenging when technology-enabled interventions were not widely used in the school. Resources to support use, including time and reminders, were highlighted, as was the need for local support, because a minimum of two engaged teachers were needed. Ideally, this would be a combination of senior and junior teachers, as the school head teacher or the special educational needs coordinator would often want to roll out the intervention but was not required to actually implement it (the class teacher was needed to implement it). Some teachers reported that they were happy to support the use of ReZone, but not necessarily promote its use due to their lack of engagement with the intervention or their busy schedules with competing priorities such as exams and other targets.

Teachers felt the facilitators of ReZone were visually engaging and that the app was interactive in nature and complimented other tools with which students were familiar. However, the lack of availability of tablet computers was at times described as a barrier. In addition, the need for teachers to be present and monitor usage limited implementation predominantly due to concerns of risk of students damaging the computers. The teachers reported availability as an important factor for fostering engagement, because if a student asked to use ReZone but the tablet computer was not available, the student was unlikely to ask again.

Parental engagement in the research was described as a barrier to implementation, especially because it led to consent forms being returned and prevented bigger groups from taking part; in addition, some schools felt an alternative approach (eg, opt-out parental consent) would increase the ease of implementation. In the mainstream schools, some teachers reported that parents specifically did not want their child using an intervention related to mental health and well-being. Moreover, teachers reported that parents/carers of students who were identified to be most in need were, at times, the hardest to engage and least likely to provide consent for the study.

Teachers reported maintaining student engagement in the app as a barrier to implementation, especially after the initial novelty had worn off. Some teachers reported that students enjoyed revisiting activities in the app, whereas other teachers thought the students wanted different stimuli and activities that offered immediate rewards each time. Similarly, some teachers reported that some students did not want to revisit situations after they had occurred and, in turn, did not want to use ReZone to reflect on the situation.

Teachers reported that some students would spontaneously discuss what they were working on with other students while using ReZone. Unsurprisingly, teachers reported that the most engaged students were the ones actively seeking help with their emotions. Moreover, teachers reported that implementing ReZone at the start of a new school year with a cohort of students was much easier than trying to integrate it as part of school routines after the year had started.

## Discussion

The aim of this study is to address the gap in evidence of the implementation of mHealth interventions. We used preliminary data and data from teacher consultations from a larger RCT conducted in schools to consider implementation barriers, facilitators, and strategies. The preliminary data include 36 students in the intervention arm across 6 schools who participated in 1082 sessions.

Implementation strategies should involve both teacher-directed and student-requested use of the tool. Feedback points to initial teacher direction for new resources, as needed, with steady integration, allowing students to independently request use over time. ReZone can be embedded into the current tools offered to students when they are feeling overwhelmed or when an incident has occurred, or as a reward/incentive or a preventative measure.

Use of the tool for the whole class can work to address the general well-being or a specific stressful period, for example, exams. Any limitations can be overcome if the school has a sufficient number of digital devices and can find time within the timetable to set aside for use of ReZone. In a class-use scenario, sessions would be less individually tailored, and there is a possibility of lower concentration and openness while discussing feelings in a group setting and more influence of social concerns.

Based on teacher guidance, implementation barrier themes seem to be widely related to the domains within the NASSS framework [23], mainly the technology, the adopter system, and the organization. A common issue raised by the consultations was related to the point of contact: The advocate for the intervention was not necessarily the one who actually implements it on a daily basis (ie, the class teacher). Due to barriers such as competing priorities, busy schedules, and engagement with the intervention, teachers could support but not promote the use of the tool. Implementation can also be impacted by staff agenda, opinions, preferences, and resistance to change routine practice. The NASSS framework and other evidence [19,20] highlight that the school system, as a whole, needs to be considered with a shared vision and plan to encompass a new technology program. School-wide capacity, readiness, and willingness are all potential barriers. These barriers are related to the feedback regarding who has the main investment in the program, emphasizing the importance of the school head being on board and active. As briefly addressed in our consultations, reluctance or unmanageable change to routine, specifically, the potential change brought on by a new technology program, is a possible barrier. Work routines can be disrupted, with a sensitive transition period when merging old and new routines. Other issues include the novelty of the technology to the school’s current system, the level of support, and the visibility of the impact of the program. Implementation of the intervention also depends on whether the school already incorporates tablets into its daily methods and routines and the level of change.

Teachers felt that technology can increase engagement through illustrations, design, interactive elements, and general appeal within the population of young people [27,28]. However, within alternative provision schools, the concern related to the risk of damage was evident and can increase reluctance to suggest tablet use in some circumstances. In mainstream schools, budgets often permit only one tablet per class or school-wide sharing of tablets. This system can be implemented using a booking system and extra planning. However, when a student asks for the tool, it may not always be available, which could be a possible deterrent for future requests of the tool. The NASSS framework highlights the importance of our methods of prototyping ReZone sufficiently, ensuring it is usable and attractive to the target user. The issue of budget allocation is also a potential barrier in the framework and was evident when some teachers discussed only having one tablet per class for use.

Parental engagement is another potential barrier: Teachers felt that some parents were difficult to engage in general communication and were therefore unlikely to sign and return letters sent home, including those for consent for their child to take part in a research study. Teachers often felt frustrated by this, as it was often these parents/carers whose children could potentially benefit from the intervention the most. Additionally, some parents were apprehensive about the level of technology their child was using and did not want them to take part in such a study. It is therefore worthwhile to attempt addressing parent/carer views, opinions, and communication preferences before the start of such a study. We worked with parents/carers on the layout and language for our information sheets and consent forms. Future work should also consider ways to increase acceptability of the research, particularly with a digital focus within this population. The NASSS framework questions what is required from those indirectly involved, such as carers. Although nothing may be required from them, if the child is using the tool at school, the question of acceptability persists.

The barriers related to students were more commonly highlighted in alternative provision schools than in mainstream schools. These barriers included opposition to repeating activities, revisiting an incident to talk through it, difficulty concentrating on a specific task, and the need for instant impact from activities. Acceptance and consideration of the work required by the adopter is a challenge considered in the NASSS framework. It can be difficult to implement long-term mental health interventions when their effect is not always immediately evident. Teacher consultations aligned with the framework in drawing attention to the level of support needed to use the technology. Most young people are familiar with the use of tablets [27] and can use them independently. However, teacher guidance and support are needed to address the initial hesitation for new things and to remember that the tool is available for use. Due to the high pupil turnover in these types of schools, attrition can be higher than that in mainstream schools.

Implementation strategies, barriers, and facilitators for digital interventions need to be considered in order to limit nonadoption and inadequate implementation in larger trials. Topics that need attention involve requirements and characteristics of the intended user group, the technology, and the organization where the tool will be implemented.

As this study was an analysis of preliminary data, the results of the full trial are needed to examine the effectiveness of ReZone. Other limitations of the present study are mentioned below. First, the consultations were conducted with a small number of teachers, and it is likely that most teachers who engaged with the study were more likely to give their views and experiences. Future research should examine implementation strategies with teachers least likely to adopt technology entirely or identify nonadopters early if implementation needs to be abandoned. Second, as with digital psychotherapy research, in general, a lack of allocation concealment and a reliance on self-report measures are also limitations.

Notwithstanding the abovementioned limitations, the findings of this research suggest that ReZone was successfully implemented and utilized, and this study identified implementation strategies, barriers, and facilitators, which are essential ingredients of larger effectiveness trials. Important considerations involve tailoring the characteristics of the intervention to the requirements of the intended user group, the technology itself, and the organization in which it is implemented.

## Abbreviations

CONSORT: Consolidated Standards of Reporting Trials
mHealth: mobile health
NASSS: nonadoption, abandonment, scale-up, spread, and sustainability
RCT: randomized controlled trial
